# Promising Immunotherapy in Metastatic Testicular Sex Cord Stromal Tumours After First-Line Chemotherapy

**DOI:** 10.3389/fimmu.2021.720359

**Published:** 2022-01-10

**Authors:** Bingqing Shang, Chuanzhen Cao, Weixing Jiang, Hongzhe Shi, Xingang Bi, Chengxu Cui, Jianzhong Shou, Shan Zheng, Jin Zhang, Aiping Zhou, Changling Li, Jianhui Ma

**Affiliations:** ^1^ Department of Urology, National Cancer Center/National Clinical Research Center for Cancer/Cancer Hospital, Chinese Academy of Medical Sciences and Peking Union Medical College, Beijing, China; ^2^ Department of Medical Oncology, National Cancer Center/National Clinical Research Center for Cancer/Cancer Hospital, Chinese Academy of Medical Sciences and Peking Union Medical College, Beijing, China; ^3^ Department of Pathology, National Cancer Center/National Clinical Research Center for Cancer/Cancer Hospital, Chinese Academy of Medical Sciences and Peking Union Medical College, Beijing, China; ^4^ Department of Radiology, National Cancer Center/National Clinical Research Center for Cancer/Cancer Hospital, Chinese Academy of Medical Sciences and Peking Union Medical College, Beijing, China

**Keywords:** testicular sex cord stromal tumours, metastasis, immunotherapy, immune checkpoint inhibitors, PD-1 inhibitor, prognosis

## Abstract

**Background:**

Testicular sex cord stromal tumours (TSCSTs) are rare, with few studies focusing on the metastatic TSCST prognosis. The value of treatments, including radical orchiectomy (RO) and retroperitoneal lymph node dissection (RPLND), in preventing metastasis is controversial. Additionally, metastatic TSCSTs are resistant to chemotherapy. We aimed to assess the effectiveness and safety of immunotherapy in metastatic TSCSTs after first-line chemotherapy.

**Methods:**

We retrospectively screened patients with testicular tumours undergoing testis surgery between January 2005 and January 2019. Patients with TSCSTs who had undergone testis-sparing surgery (TSS) or RO were identified. The malignant type was defined as metastasis confirmed by pathology. Treatment responses, progression-free survival (PFS), overall survival (OS) and safety were analysed.

**Results:**

Among the 494 testicular tumour patients who received TSS or RO, 11 (2.2%) patients with histologically proven TSCSTs were identified. At the last follow-up, 7 patients survived without tumours, and 4 patients developed metastasis and received first-line cisplatin-based chemotherapy, with 1 of them achieving an objective response. Their PFS times were 1.5, 2.2, 9.0, and 17.0 months, respectively. Two patients received immune checkpoint inhibitors (ICIs) after developing chemotherapy resistance and achieved a partial response up to the last follow-up; one of them experienced Grade 1 adverse events, and the other experienced Grade 2 adverse events during immunotherapy. The median OS time of the 4 patients with metastatic TSCSTs was 32 months.

**Conclusions:**

TSCSTs are rare, and most are benign with a good prognosis. ICIs represent a promising option for improving clinical outcomes in metastatic TSCSTs.

## Introduction

Testicular tumours are classified as germ cell tumours or non-germ cell tumours. Testicular sex cord stromal tumours (TSCSTs) are the most common type of testicular non-germ cell tumour. However, TSCSTs have a low incidence rate, accounting for only 5% of testicular tumours (1, 2). The pathological types of TSCSTs include Leydig, Sertoli, and other types. Radical orchiectomy (RO) is the main treatment for TSCSTs, and testis-sparing surgery (TSS) can be considered for patients with small tumours. Previous studies have reported that the prognosis of patients with benign TSCSTs is good; however, approximately 10%–12% of TSCSTs are malignant (3–5). Some patients present with metastases at the first visit, and others are found to have metastases during follow-up after testicular surgery. The median time of metastasis is approximately 12–28 months (4, 5). The most common sites of metastases are the retroperitoneal lymph node, lung, bone, and liver (3–5). Metastatic TSCSTs are not sensitive to radiotherapy and chemotherapy, and there is no consensus on treatment (2, 6, 7). Compared with that of testicular germ cell tumours, the prognosis of metastatic TSCSTs is poor, and the 5-year survival rate is less than 24.2% (8). In particular, for patients with rapid progression, platinum-based chemotherapy or palliative symptomatic treatment can be used only to improve quality of life.

In the era of immunotherapy, immune checkpoint inhibitors have become the focus of cancer treatment. Inhibitors of the most representative programmed death 1 (PD-1) and its ligand PD-L1 (PD-1/PD-L1) are new options for advanced malignant tumours. PD-1/PD-L1 inhibitors alone or in combination with targeted therapy can significantly improve the survival of patients with advanced non-small-cell lung cancer (9), melanoma (10), and renal cell carcinoma (11). However, due to the low incidence of malignant TSCSTs, no study has reported the efficacy of PD-1/PD-L1 inhibitors in this setting. Therefore, here, we summarize the clinicopathological features and prognosis of TSCSTs and, for the first time, explore the efficacy of PD-1 inhibitors in patients with metastatic TSCSTs after failure of first-line chemotherapy. The results are reported below.

## Patients and Methods

We retrospectively screened patients with testicular tumours undergoing testis surgery at the National Cancer Center/Cancer Hospital, Chinese Academy of Medical Sciences (NCC/CHCAMS), between January 2005 and January 2019. Among 494 testicular tumour patients, 11 (2.2%) patients with histologically proven TSCSTs were identified and included in this study. The medical records of the identified patients were retrospectively reviewed to collect the relevant demographic data (mean age at surgery), clinical data (main symptoms, physical examination and laboratory examination), tumour characteristics, and oncological outcomes [vital status, progression-free survival (PFS), overall survival (OS)]. All the data were anonymized for analysis. Staging was performed by computed tomography (CT) scans.

All patients underwent inguinal access to the testis, but because of the heterogeneous origin of the cases and the long period of observation, it was not possible to report a univocal set of indications regarding the extent of surgery (i.e., RO vs. TSS). At our institution, intraoperative frozen section examination during testicular surgery is currently mandatory, with cases of bulky tumours excluded, whereas a TSS is attempted in cases of tumours not exceeding one-third of the testis volume or with fertility demand.

Circulating tumour DNA (ctDNA) was extracted for sequencing from two patients before receiving PD-1 immunotherapy treatment to evaluate tumour mutation burden (TMB) and microsatellite instability (MSI) status. An unstained slide was obtained from the formalin-fixed paraffin-embedded (FFPE) tumour of one patient. PD-L1 expression was identified by immunohistochemical (IHC) by staining with the PD-L1 IHC 22C3 pharmDx kit.

Patients were followed up every 3–6 months for the first 2 years postoperatively and annually thereafter. Prognosis information was obtained through telephone follow-ups and the Electronic Medical Record System. Follow-up evaluation consisted of physical examination, laboratory tests, and imaging examination information on treatments after surgery, time of recurrence or metastasis, cause and time of death. All follow-ups were concluded on January 31, 2021.

Clinical parameters at the time of initial metastasis were classified in terms of the 8th American Joint Committee on Cancer (AJCC) tumour-node-metastasis (TNM) staging system. Homologous metastasis was confirmed by biopsy or resection of metastases. Tumours were assessed by CT or magnetic resonance imaging (MRI) at baseline and every 2 treatment cycles. Tumour response and disease progression were documented using the Response Evaluation Criteria in Solid Tumours (RECIST) criteria. Overall objective response (ORR) includes complete response (CR) and partial response (PR). Detection and tracking of adverse events were carried out according to the Common Terminology Criteria for Adverse Events (CTCAE). PFS was defined as the time from the date of randomization to the first date of documented progression or death as a result of any cause. If no PFS event was observed, PFS was censored at the date of the last evaluable tumour assessment. OS was defined as the time from the date of randomization to the date of death due to any cause. To clearly illustrate this information, we adopted a swimmer plot to capture the time of response onset and duration of response when a therapy was given. Patient consent was assigned to each medical record.

## Results

**Figure 1:**
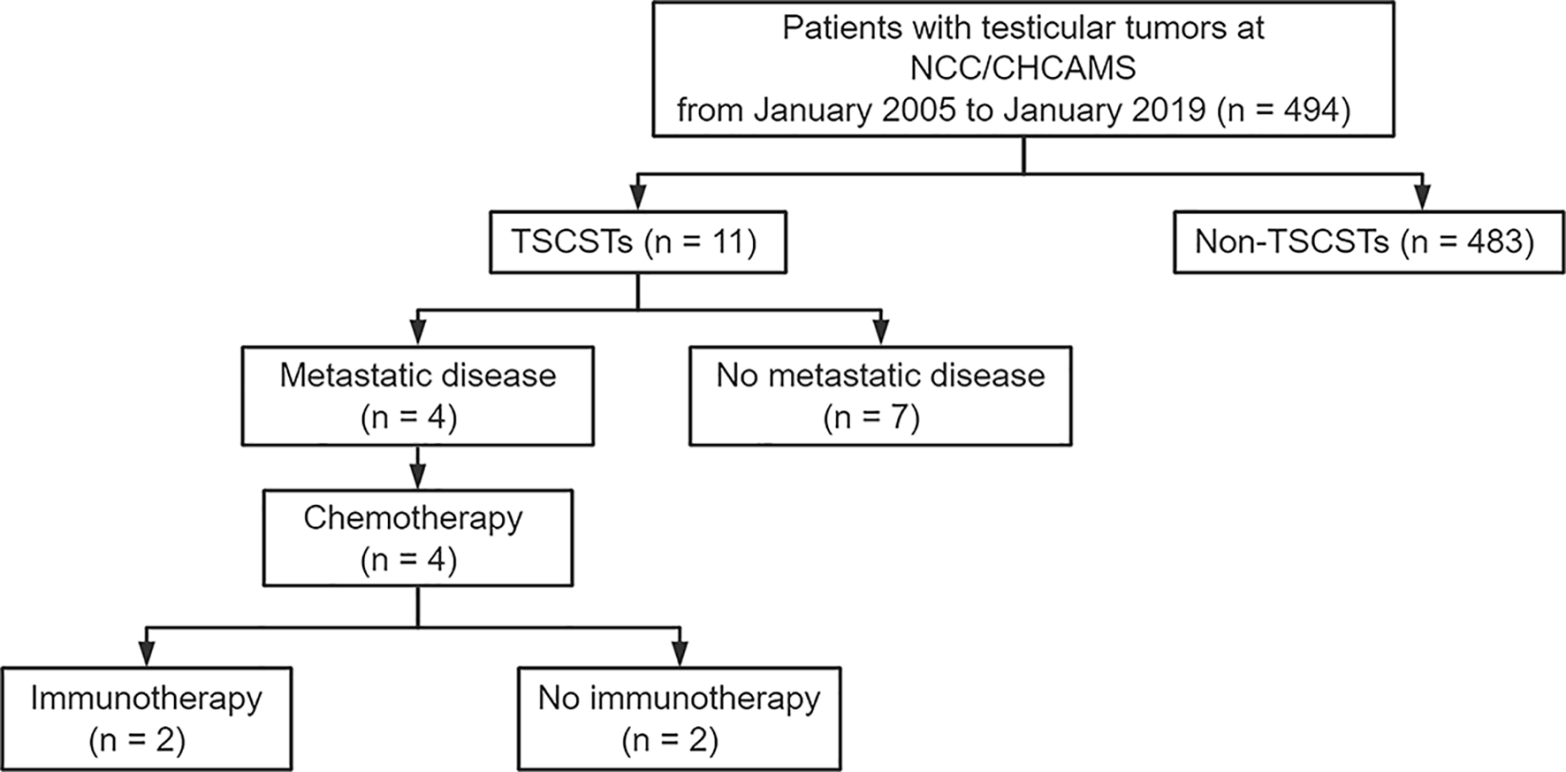
Flowchart of screening patients. NCC/CHCAMS, National Cancer Center/Cancer Hospital, Chinese Academy of Medical Sciences; TSCSTs, testicular sex cord stromal tumours.

Among the 494 testicular tumour patients, 11 (2.2%) patients with histologically proven TSCSTs, including 9 patients with Leydig cell tumours and 2 patients with Sertoli cell tumours, were identified and included in this study. The flowchart for patient screening is shown in **Figure 1**. The median age was 36 years (range, 18–71 years), the median tumour size was 3.8 cm (range, 0.8–7.0 cm), the median alpha fetoprotein (AFP) concentration was 2.82 ng/ml (range, 1.8–3.43 ng/ml), the median beta-human chorionic gonadotropin (β-HCG) concentration was 0.1 mIU/ml (range, 0.1–1.81 mIU/ml), the median lactate dehydrogenase (LDH) concentration was 175 U/l (range, 118–185 U/l), and the median testosterone (T) concentration was 15.09 nmol/l (range, 10.15–18.94 nmol/l). The levels of all these endocrine parameters were within the normal range. The haematoxylin–eosin staining results of Leydig cell tumours and Sertoli cell tumours are shown in **Supplementary Figures S1A, B**. The characteristics of the 11 TSCST patients are summarized in **Table 1**.

**Table 1:** Clinical characteristics of 11 patients of testicular sex cord stromal tumours from January 2005 to January 2019.

Patient	Age	Side	Tumour size (cm)	AFP (ng/mL)	β-HCG (mIU/mL)	LDH (U/L)	T (nmol/L)	Surgery	Surgery time	Pathological features	Follow-up time*^a^* (m)	Status
1	18	Left	7	2.79	0.1	145	NA	RO	2005.7	Leydig	189	Alive
2	36	Left	0.8	3.08	0.1	118	NA	TSS	2007.1	Leydig	171	Alive
3	24	Left	2.7	2.95	1.81	185	NA	RO	2008.9	Leydig	151	Alive
4	33	Right	1.1	2.49	0.1	178	10.15	TSS	2009.1	Leydig	147	Alive
5	71	Left	2	1.8	0.1	133	12.02	TSS	2009.8	Leydig	140	Alive
6	53	Left	6	2.97	0.1	175	13.86	RO	2010.6	Leydig	76	Dead
7	33	Left	5	3.43	0.1	172	18.94	RO	2010.9	Leydig	8	Dead
8	42	Right	3.8	2.14	0.196	176	15.21	RO	2011.7	Leydig	115	Alive
9	40	Right	0.9	2.82	0.1	183	15.39	TSS	2011.9	Sertoli	113	Alive
10	28	Left	5.6	2.85	0.14	134	16.48	RO	2018.4	Sertoli	34	Alive
11	48	Left	7	2	0.1	177	14.96	RO	2018.6	Leydig	32	Alive

^a^The follow-up time was concluded to January 31, 2021.

AFP, alpha fetoprotein; β-HCG, beta-human chorionic gonadotropin; LDH, lactate dehydrogenase; RO, radical orchiectomy; T, testosterone; TSS, testis sparing surgery.

**Figure 2:**
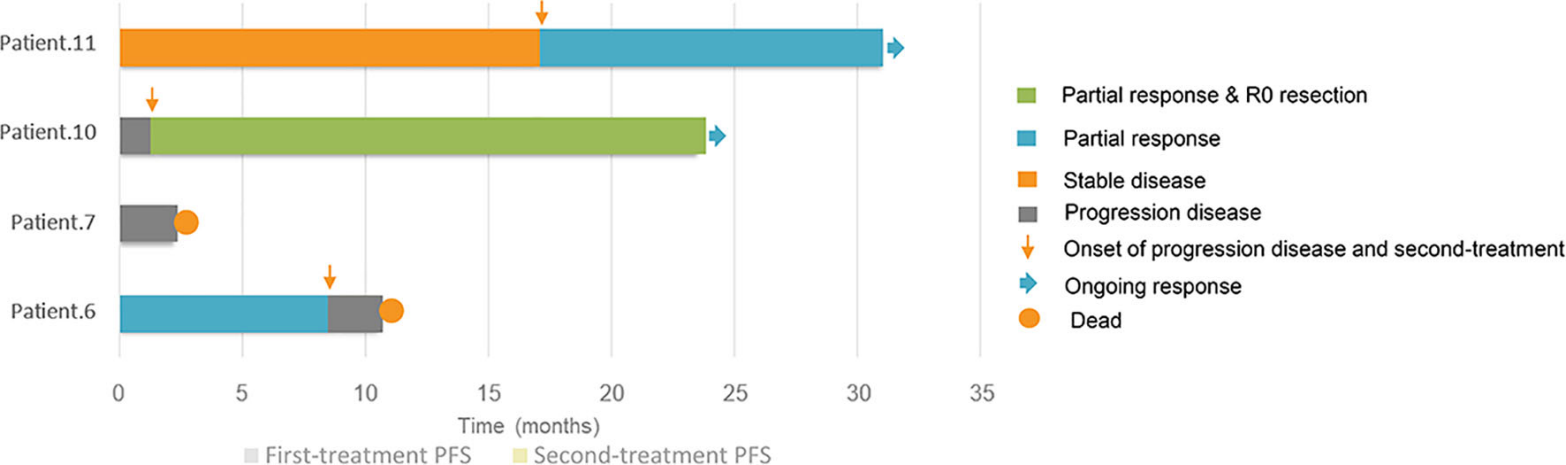
Swimmer plots of progression-free survivals and responses of first-line and second-line treatment for 4 patients of metastatic testicular sex cord stromal tumours.

**Figure 3:**
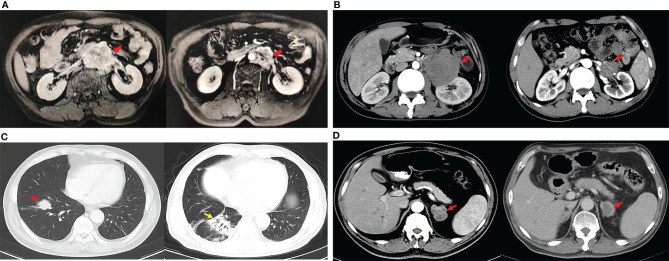
Changes of metastatic lesions during treatments. **(A)**. PR of the patient after first-line chemotherapy (paclitaxel and cisplatin): the maximum diameter of the retroperitoneal lymph node metastasis (red arrows) decreased from 7.4 cm (left) to 4.4 cm (right). **(B)** The enhanced CT showed the shrinkage of the retroperitoneal metastasis (red arrows) after sintilimab plus anlotinib. The maximum diameter of the lesion decreased from 8.2 cm (left) to 2.6 cm (right) considering partial response. **(C)** The enhanced chest CT showed the metastasis located at the right lung (red arrow) disappeared after PD-1 antibody, but interstitial pneumonia appeared (yellow arrow). **(D)** Meanwhile, the maximum diameter of the left adrenal metastasis (red arrows) decreased from 3.6 cm (left) to 2.8 cm (right) of the same patient described in **(C)**, so the partial response was considered.

The median follow-up time was 140.0 months (range 8.0–189.0), and 63.6% (7/11) of patients were considered to have benign TSCSTs with no metastasis up to the follow-up date. The prognosis was favourable, and the OS range was 113.0–189.0 months. However, the other 4 (36.4%) patients had metastasis during follow-up and were diagnosed with malignant TSCSTs. The metastasis times were 1.0, 1.2, 9.0, and 48.0 months. The sites of metastasis were the retroperitoneal lymph node, adrenal gland, and lung. Metastatic TSCST cases had significantly larger primary tumours (5.8 vs. 2 cm, p = 0.021) and were more likely to present with any adverse factor (necrosis, angiolymphatic invasion, pleomorphism, high mitotic index, atypia) (75% vs. 0%, p = 0.007) (**Table 2**). Cisplatin-based chemotherapy was performed in cases of metastatic TSCSTs. The PFS times of first-line chemotherapy were 1.5, 2.2, 9.0, and 17.0 months (**Table 3** and **Figure 2**). Among them, one patient achieved PR. This patient developed retroperitoneal lymph node metastases, the largest of which was 7.4 cm (**Figure 3A**, left). The regimen of paclitaxel plus cisplatin was performed every 3 weeks for 8 cycles. After chemotherapy, MRI showed that the retroperitoneal metastases shrank and that the largest metastases decreased to 4.4 cm (**Figure 3A**, right), indicating that PR was reached. Nevertheless, new lung metastases occurred, and the disease progressed soon after.

**Table 2:** Clinical and pathological features of metastatic and non-metastatic TSCSTs.

	Metastatic TSCSTs, n = 4 (%)	Non-metastatic, n = 7 (%)	p-value
**Age (years)**			0.781
Median (IQR)	40.5 (28–53)	36 (18–71)	
≤42 years	2 (50)	6 (86)	
>42 years	2 (50)	1 (14)	0.201
**Size (cm)**			0.021
Median (IQR)	5.8 (5–7)	2 (0.8–7)	
≤3 cm	0 (0)	5 (71)	
>3 cm	4 (100)	2 (29)	0.022
**Adverse risk factors*^a^***			0.007
None	1 (25)	7 (100)	
1 or more	3 (75)	0 (0)	

^a^Necrosis, angiolymphatic invasion, pleomorphism, high mitotic index and atypia were included in adverse risk factors.

TSCSTs, testicular sex cord stromal tumours.

**Table 3:** Treatments and survivals of 4 patients with metastatic testicular sex cord stromal tumours.

Patient	Time of metastasis after operation (m)	Location of metastasis	Stage	Pathological features	First-line treatment	Response of first-line treatment	First-line PFS (m)	Second-line treatment	Response of second-line treatment	Second-line PFS (m)	OS (m)	Follow-up time (m)
6	48	Retroperitoneal lymph node	II	Leydig	Paclitaxel and cisplatin	PR	9	Epirubicin and cyclophosphamide	PD	1.5	76	76
7	1.2	Retroperitoneal lymph node, lung	III	Leydig	Paclitaxel and cisplatin	PD	2.2	NA	NA	NA	8	8
10	9	Retroperitoneal lymph node	II	Sertoli	Gemcitabine and cisplatin	PD	1.5	PD-1 inhibitor and TKI; RPLND	PR; R0 resection	22*^a^*	34*^a^*	34*^a^*
11	1	Right lung, left adrenal	III	Leydig	Paclitaxel and cisplatin	SD	17	PD-1 inhibitor	PR	14*^a^*	32*^a^*	32*^a^*

^a^The survival status of two patients was survival and the tumour had not progressed; the follow-up time was concluded to January 31, 2021.

OS, overall survival; PD, progression disease; PFS, progression-free survival; PR, partial response; RPLND, retroperitoneal lymph node dissection; SD, stable disease.

After developing chemotherapy resistance, 2 patients received PD-1 inhibitor immunotherapy alone or in combination with targeted therapy. One patient of Sertoli cell tumour with retroperitoneal lymph node metastasis (**Figure 3B**, left) received sintilimab (200 mg) intravenously once every 3 weeks combined with anlotinib (12 mg), a novel multitargeted tyrosine kinase inhibitor (TKI), orally once daily on days 1–14 of a 21-day cycle for 4 cycles. After treatment, the retroperitoneal metastasis shrank, and PR was reached (**Figure 3B**, right). Then, R0 resection by retroperitoneal lymph node dissection (RPLND) was performed. Another patient of Leydig cell tumour with right lung metastasis (**Figure 3C**, left) and left adrenal metastasis (**Figure 3D**, left) received sintilimab (200 mg) intravenously once every 3 weeks of monotherapy beginning immediately after first-line chemotherapy had failed. After treatment, the right lung metastasis disappeared (**Figure 3C**, right), and the left adrenal metastasis shrank (**Figure 3D**, right), indicating that PR was reached. Sintilimab alone or combined with TKI therapy improved the prognosis of two patients. The PFS times were 14 months and 22 months, and these 2 patients were still in follow-up (**Table 3**, **Figure 2**).

According to the tumour molecular profiles of two patients receiving immunotherapy with or without TKI, one patient of sertoli cell tumour showed a low TMB with 2.00 mutations/Mb of sequenced DNA, with microsatellite stable status. The TMB was also low as 3.23 mutations/Mb in another patient of Leydig cell tumour, with a baseline biopsy indicating <1% PD-L1+ cells in the tumour.

During first-line chemotherapy, adverse reactions were evaluated as follows: myelosuppression manifesting as Grade 3 leukopenia, as well as Grade 2 dyspepsia. During immunotherapy, one patient experienced Grade 2 interstitial pneumonia, and another patient treated with immunotherapy plus targeted therapy experienced Grade 1 liver dysfunction and fever. No severe adverse events (Grade 3/4/5) occurred during immunotherapy.

## Discussion

TSCSTs are rare, and relevant studies, especially for malignant TSCSTs and relevant treatments, are few. For the first time, we have summarized the clinicopathological features and prognosis of TSCSTs in our centre and explored the efficacy of PD-1 inhibitors in patients with metastatic TSCSTs after failure of first-line chemotherapy. The ages of the TSCST patients ranged from 2 to 90 years, and the median onset age was approximately 45 years (4, 12). In our study, the median age was 36 years, which was lower than that in previous studies. This may be attributable to early disease detection. The incidence rate of Leydig cell tumours in the previous study was approximately 3% (4), while that of Sertoli cell tumours was 0.4%–1.5% (13). Similarly, in our study, the incidence rate of Leydig cell tumours was approximately 1.8% (9/494), and that of Sertoli cell tumours was approximately 0.4% (2/494). Among these patients, 63.6% (7/11) had benign tumours and favourable survival outcomes. The OS times ranged from 113.0 to 189.0 months up to the follow-up date, and the patients survived without tumours.

Malignancy of the TSCST is related to older age, larger tumours, and aggressive pathological features, including necrosis, angiolymphatic invasion, pleomorphism, a high mitotic index, and atypia (14). In our study, apart from age, other clinical and pathological features were consistent among our patients (**Table 2**). However, these features were not sufficient for a complete prediction of the outcome. The only reliable standard depended on the appearance of metastases. Some patients presented with metastases at the first visit, and others were found to have metastases during follow-up. The median time of metastasis was 12–28 months (4, 5). One patient was found to have metastasis up to 8 years after orchiectomy (15). The most common location of metastasis was the retroperitoneal lymph node, lung, bone, and liver. In this study, 36.3% (4/11) of patients experienced metastasis. One patient had metastasis at the first visit, and the other three patients had metastasis during the follow-up. The metastasis times of the four patients were 1.0, 1.2, 9.0, and 48.0 months.

For metastatic TSCSTs, it has been reported that RPLND could be curative in patients with a single retroperitoneal lymph node metastasis (16). In our study, there was also one such patient who underwent R0 resection with RPLND after immunotherapy. In a previous retrospective study, eight patients with stage II and stage III metastatic TSCSTs who received RPLND had a median OS of 14.4 months despite aggressive surgical treatment (abdominal and pelvic mass resection, enterotomy, hepatectomy, and splenectomy) and additional postoperative treatment (one patient received radiotherapy, and five patients received cisplatin-based chemotherapy) (17). It is thus clear that for patients with malignant TSCSTs, especially those with multiple metastases, the effect of surgical treatment is poor, the disease progresses rapidly, and the prognosis is extremely poor. Previous studies indicated that chemotherapy for malignant TSCSTs, such as bleomycin, cisplatin, and etoposide (BEP), cisplatin, vinblastine, and bleomycin (PVB), etoposide and cisplatin (EP), and vinblastine, ifosfamide, and cisplatin (VIP), was not effective (17–19). Similarly, targeted drugs such as imatinib and brentuximab (an antibody drug targeting CD30) also showed no effect in the treatment of TSCSTs (20, 21). One patient in this group achieved PR after paclitaxel and cisplatin combined chemotherapy; however, the remission period was short, and disease progressed immediately after the end of chemotherapy.

Therefore, the progression of malignant TSCSTs is rapid, and there is an urgent need to identify an effective treatment. Among some of the candidates, PD-1/PD-L1 inhibitors have been shown to significantly improve the survival rate of patients with advanced cancer. Necchi et al. explored the genomic changes in malignant TSCSTs and found that the microsatellite instability mutation rate is low and that it has the characteristics of a low TMB. Thus, they speculated that the response to immunotherapy would be limited (22). However, Aguilar et al. found that the growth rate of TSCSTs in mice could be significantly decreased by injecting an inhibin-α vaccine in advance. They suggest that the immune response plays an important role in its anti-tumour activities, but the mechanism is unclear (23). Therefore, the expected effect of immunotherapy on TSCSTs is controversial. This study is the first to evaluate the clinical efficacy of PD-1 inhibitor immunotherapy in patients with metastatic TSCSTs. Two patients with metastatic TSCSTs showed a clear objective response to PD-1 immunotherapy. The PFS times of the first and second patients were above 14 and 22 months, respectively, and they are still under follow-up. The median OS time of 4 patients with metastatic TSCSTs was 32 months. In previous reports, the prognosis of advanced TSCSTs was poor, with a median OS of approximately 24 months (17, 24). At the same time, the two patients did not have any Grade III, IV, or V adverse reactions during PD-1 inhibitor immunotherapy. We report the efficacy and toxicity of PD-1 inhibitors for metastatic TSCSTs for the first time. What is more, we evaluated the molecular characteristics of metastatic TSCSTs. Consistent with the prior study, metastatic TSCSTs featured with low TMB and low MSI status in tumour (22). The possible reason is that there are many factors affecting the efficacy of immunotherapy for metastatic TSCSTs, which needs comprehensive evaluation, and cannot rely on PD-L1, MSI, or TMB alone.

This study had some limitations, including its retrospective and single-centre design. However, the use of consecutive patients was an attempt to mitigate some of the deficiencies, and the national cancer centre could have a certain representativeness. At present, there are few reports about immunotherapy for metastatic TSCSTs, and its management is still challenging. Due to the rarity of the disease, we used PD-1 inhibitors in only 2 patients.

In conclusion, TSCSTs are rare, and most are benign with a favourable prognosis. However, traditional therapies have a poor response for malignant TSCSTs. We explored the efficacy of immunotherapy for malignant TSCSTs for the first time, and the results were promising. Further studies are needed to explore its value as a first-line regimen.

## Data Availability Statement

The original contributions presented in the study are included in the article/**Supplementary Material**. Further inquiries can be directed to the corresponding authors.

## Ethics Statement

The studies involving human participants were reviewed and approved by the Ethics Committee of National Cancer Center/National Clinical Research Center for Cancer/Cancer Hospital, Chinese Academy of Medical Sciences, and Peking Union Medical College. The patients/participants provided their written informed consent to participate in this study. Written informed consent was obtained from the individual(s) for the publication of any potentially identifiable images or data included in this article.

## Author Contributions

CCu and JS had full access to all the data in the study and take responsibility for the integrity of the data and the accuracy of the data analysis. Concept and design: BS, CCa. Acquisition of data: BS, CCa, WJ, HS, SZ, CCu, JZ. Drafting of the manuscript: BS, CCa. Critical revision of the manuscript for important intellectual content and supervision: CL, JM, AZ, CCu, and JS. Statistical analysis: BS, CCa. Administrative, technical, or material support: BS, CCa, HS, XB, CCu, JZ, SZ. Data analysis: BS. Supervision: CCu and JS. All authors contributed to the article and approved the submitted version.

## Conflict of Interest

The authors declare that the research was conducted in the absence of any commercial or financial relationships that could be construed as a potential conflict of interest.

## Publisher’s Note

All claims expressed in this article are solely those of the authors and do not necessarily represent those of their affiliated organizations, or those of the publisher, the editors and the reviewers. Any product that may be evaluated in this article, or claim that may be made by its manufacturer, is not guaranteed or endorsed by the publisher.
