# Supplementary material for: Promising Immunotherapy in Metastatic Testicular Sex Cord Stromal Tumours After First-Line Chemotherapy
Source: Front Immunol. 2022 Jan 10;12:720359. doi: 10.3389/fimmu.2021.720359 (PMC8784380; doi:10.3389/fimmu.2021.720359)

Supplementary Material

# Supplementary Data

**Figure s1.** A. Leydig cell tumor cells arranged in sheets with abundant granular eosinophilic cytoplasm and prominent pleomorphic nuclei (H&E, 400x).


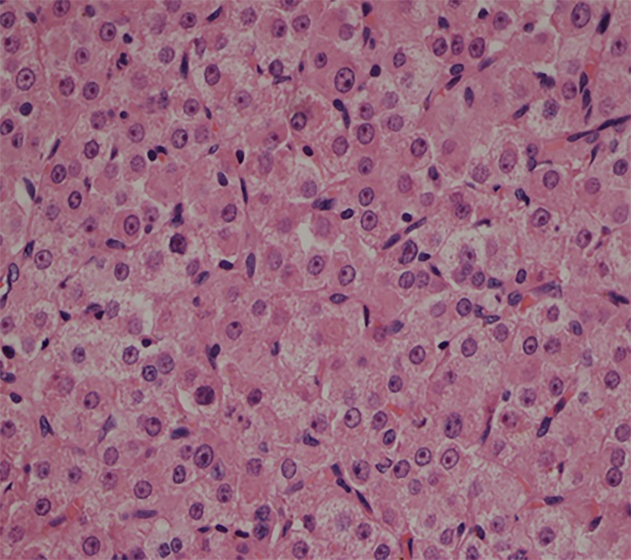


B. Sertoli cell tumor presented medium-sized columnar cells arranged in irregular cellular aggregates. The cytoplasm was abundant and contained a large amount of lipids (H&E, 400x).


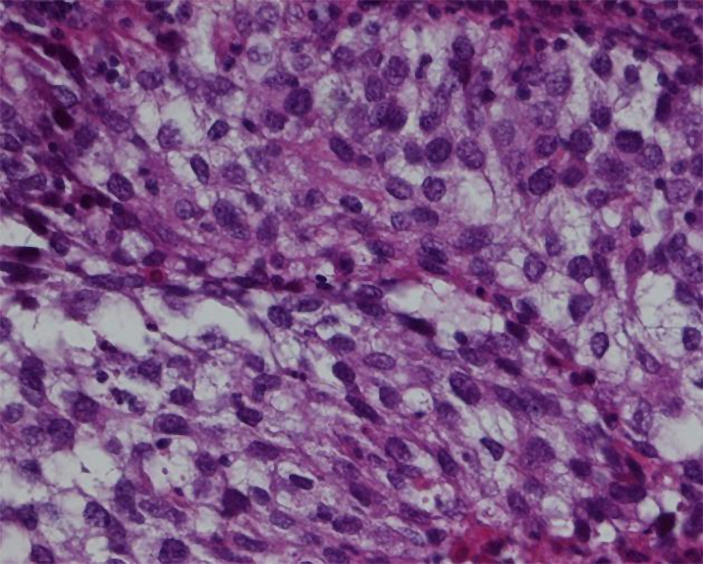

Supplement: Supplementary file 1 [file DataSheet_1.docx]
